# Evaluation of a 3D printed training model with realistic spatial-anatomical conditions for head and neck microsurgery

**DOI:** 10.1007/s00784-025-06314-4

**Published:** 2025-04-08

**Authors:** Manuel Olmos, Joy Backhaus, Rainer Lutz, Christopher-Phillip Nobis, Sarah Koenig, Marco Kesting, Manuel Weber

**Affiliations:** 1 Department of Oral and Cranio- Maxillofacial Surgery, Friedrich-Alexander-Universität Erlangen-Nürnberg, Erlangen, Germany; 2 Friedrich-Alexander-Universität Erlangen-Nürnberg (FAU), Erlangen, Germany; 3 Institute of Medical Teaching and Medical Education Research, University Hospital of Würzburg, Würzburg, Germany

**Keywords:** Simulation, Microsurgery, Teaching model, Head and neck surgery, Dentistry, 3D

## Abstract

**Objectives:**

Although existing microsurgical models provide a high degree of realism in tissue properties, they often neglect the complex and constrained spatial-anatomical conditions typical of head and neck surgery. This study aims to evaluate the effectiveness of the Head and Neck Realistic Anatomical Condition Experience (RACE) model in enhancing microsurgical education.

**Methods:**

Using a microsurgical competency assessment tool and self-assessment questionnaires, the head and neck RACE model was evaluated through application in two student courses (10 participants) and one resident course (5 participants). In both groups, first the conventional chicken thigh model and then the RACE model were applied. Data were analyzed using a two-way repeated measures ANOVA with Welch’s statistics to assess differences between the groups.

**Results:**

In pregraduate courses, the transition from the conventional chicken thigh model to the RACE model initially led to a decline across all eight microsurgical performance parameters (Q1.1-Q4.2). However, after an additional day of training with the RACE model, all parameters—except tissue-preserving technique (Q1.2) — returned to or significantly exceeded baseline levels (Q1.2 *p* = 0.373, Q1.3 *p* = 0.003, Q2.1 *p* < 0.001, Q2.2 *p* = 0.022, Q2.3 *p* = 0.008, Q3.1 = 0.014, Q4.1 *p* = 0.036, Q4.2 *p* = 0.002). Conversely, residents showed immediate improvement in all parameters, except for suture distance to the vessel’s margin, upon switching to the RACE model.

**Conclusions:**

Head and neck RACE models provide a challenging and practical addition to microsurgery teaching.

**Clinical relevance:**

The positive impact on learning outcomes in this area supports the development of RACE models in other areas of microsurgical and general medical training, and therefore the education of students and clinical practitioners.

**Supplementary Information:**

The online version contains supplementary material available at 10.1007/s00784-025-06314-4.

## Background

The first documented use of simulation in surgical training dates back to 600 BC, when leaf and clay models were used to teach the “Indian forehead flap” [1]. Today, simulation is firmly established in the training of pilots, astronauts and soldiers, while medical training has only recently ceased Halsted’s teaching model (“See one, do one, teach one“) in favor of simulation-based learning [2–8].

Underlining the importance of teaching in microvascular surgery is the fact that the training and experience of the operating microsurgeon was identified across studies as the main factor in the success of microsurgical tissue transfer [9–11]. Current microsurgical teaching models can be broadly categorized into synthetic, non-living animal and living animal models, with their respective advantages and limitations [12–16]. In comparison and based on a modified classification of the Oxford Centre for Evidence-Based Medicine, cadaveric animal models received the highest level of recommendation regarding realism and cost effectiveness compared to other modalities [15]. Although realistic in simulation, ethical concerns have been raised about the use of living animal models [17]. Recently, non-biological simulators are gaining increasing recognition as they overcome the various ethical, financial and accessibility issues associated with the use of live or cadaveric animal models [18, 19]. Most of them can be categorized as low-fidelity models, with virtual reality models, although promising, not yet offering the haptic experience required for learning microsurgical procedures [15, 18].

Microsurgery in the head and neck area confronts surgeons with unique challenges. Both the spatial configuration of the oral cavity and the jaws as well as the larynx and the resulting submerged areas make it difficult to perform microvascular anastomoses in the head and neck region. These unique anatomical conditions, combined with high standards of patient care, require high quality training for surgeons [20]. As Nanji et al. and Mills et al. have found, the realism of a scenario depends on whether a strong link is established between the simulated situation and learners’ perceptions of fidelity and reality, with high levels of realism implying greater learner engagement [21–23].

A careful and pragmatic evaluation must confirm the applicability, realism and relevance of each training model following its development. For more than one hundred years rating scales have been used in psychology and psychophysics and are now widely used in all medical fields for measuring qualitative variables. Several types of rating scales exist in which the possible answers are either continuous or discrete. Recently, Elisabeth Svensson introduced a rank-invariant approach for inter-scale comparison to evaluate the order consistency between the visual analogue scale, the graphic rating scale and a five-point verbal descriptor scale. She showed that both the verbal descriptor scale and the graphic rating scale were superior to the visual analogue scale assessments, and that assessments on the discrete scale had the highest level of stability [24]. In line with this statement, the following evaluation of the head and neck RACE model focuses on discrete verbal descriptor scales and graphical rating scales in order to clarify the benefits and applicability of the model in microsurgical training.

Does the more realistic and therefore more challenging anatomical configuration of the head and neck RACE model initially impair students’ performance and, if so, what are the consequences of further training on the model for students’ performance?

Aim of the current analysis was to evaluate the impact of the RACE model on microsurgery in the training of residents and medical students in maxillofacial surgery based on previously established assessment parameters of microsurgical performance [25, 26].

## Methods

### Model description

**Fig. 1:**
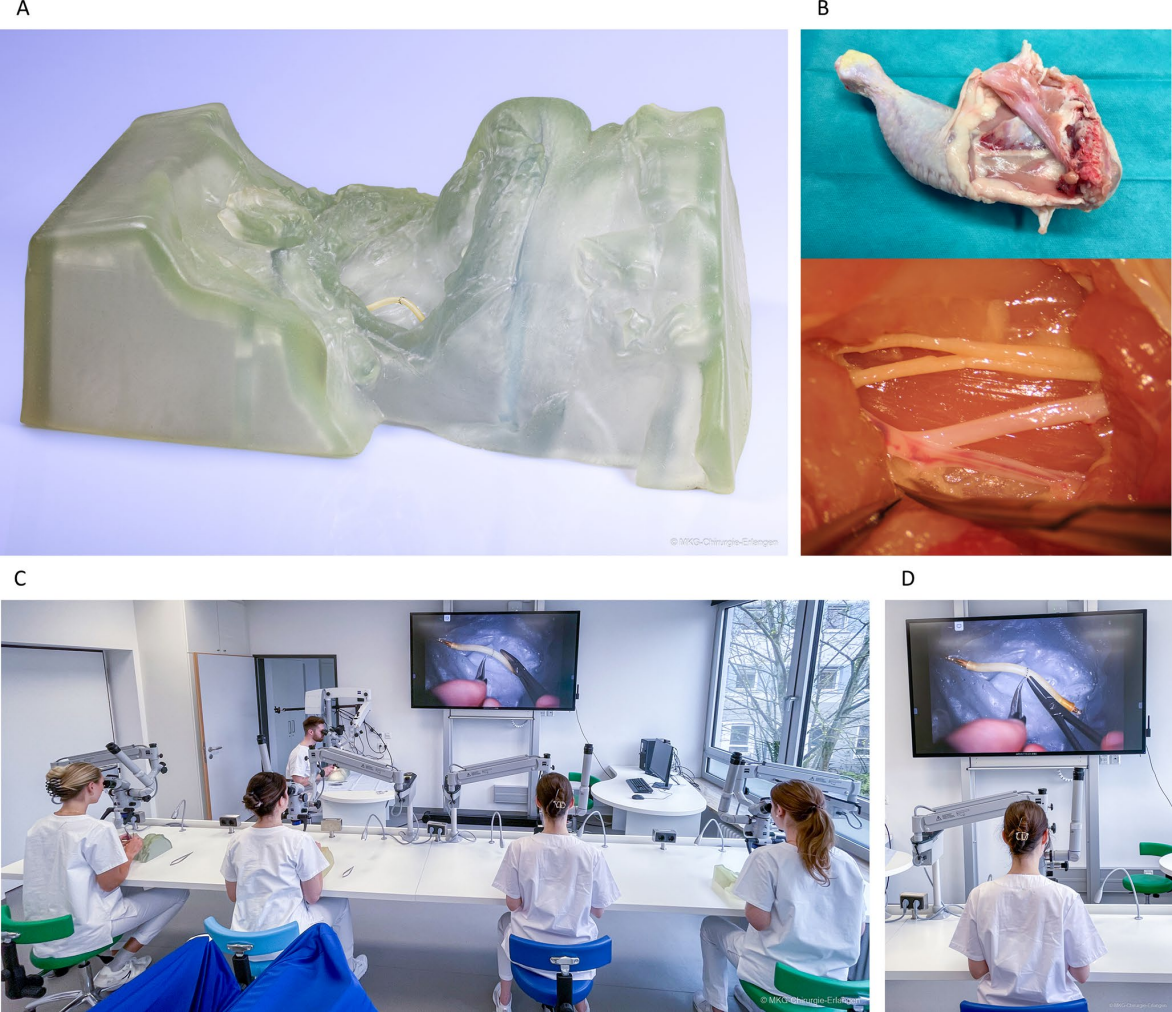
The 3D printed head and neck RACE model and its application in a pregraduate course. **(a)** Overview of the 3D-printed head and neck RACE model simulating a situation after neck dissection and prior to the microanastomosis. Microsurgical vessel connection to the superior thyroid artery is demonstrated and taught under realistic spatial-anatomical conditions. **(b)** Application of the RACE model in a course for pregraduate medical students, which takes place in the microsurgical SkillsLab of the Department of Oral and Cranio-Maxillofacial Surgery at Erlangen University Hospital. Realistic spatial-anatomical conditions (RACE) allow for simulation of microanastomosis suturing in complex surroundings

The head and neck realistic anatomical condition experience model (RACE model, Fig. 1A) was developed as a non-biological, open-source, high-fidelity simulator to translate those challenging conditions into a training situation and enable learning under realistic spatial-anatomical conditions [8]. In this case, realistic spatial-anatomical conditions describe a training environment in which structures (muscles, arteries, etc.) are located in exactly the same place throughout the entire training environment as they would be under real surgical conditions on the patient. As a result, this also allows for exact simulation of ergonomics and posture during the suturing process. Following the Principle of 3Rs (“replace, reduce, refine”) it presents an in-house 3D printable and sustainable addition to microsurgical teaching [7]. After an intraoperative surface scan of a surgical site prior to cervical microvascular anastomosis, 3D data were exported and processed using simple 3D modelling operations such as smoothing and extrusion (Meshmixer version 3.5.474, Co. Autodesk, San Francisco, California, US). Once the model was created, it was printed using stereolithography and cured / post-processed according to the 3D printer manufacturer’s guidelines (Form 3BL, Formlabs, Somerville, Massachusetts, US).

The conventional chicken model used in both the medical student and resident courses consists of a cadaveric chicken thigh in which the femoral vessels are surgically dissected and then used for microvascular anastomosis (Fig. 1B) [15].

### Participants

Initially, 2 consecutive courses with 5 students each were evaluated in the current study. A total of *n* = 40 course days were analyzed. Subsequently, the microsurgical training of 5 oral and maxillofacial surgery residents was evaluated on both course days, *n* = 10 each. The final sample size was set at *n* = 50 course days for statistical evaluation.

### Course design

8 workstations with microscopes incl. monocular co-observers (OPMI pico, Co. Zeiss, Jena, Germany) and a master microscope with 4k transmission to a demonstration screen form the centerpiece of the “Microsurgical Skill Lab” at the Department of Oral and Cranio-Maxillofacial Surgery in Erlangen (Fig. 1C and D). The RACE model was first evaluated in a microsurgical elective course for medical students. The course ran for 6 weeks with one two-hour day per week. Day 1 of the course taught basic microsurgical suturing techniques and gave participants the opportunity to familiarize themselves with the instruments and microscopes using a simple benchtop model. Day 2 was dedicated to developing a tactile feel for the tensile and tear strength of delicate fabrics through suturing exercises on peeled oranges or other citrus fruits. On course days 3 and 4, special microsurgical techniques including vascular and nerve microsurgical end-to-end anastomoses were taught by means of a conventional ex vivo chicken thigh model. If individual students made rapid progress, there was an additional opportunity to learn vascular microsurgical end-to-side anastomoses. Having learnt the microsurgical techniques, days 5 and 6 of the course focused on applying them under realistic spatial-anatomical conditions using the head and neck RACE model [8].

Additionally, an internal training course for residents in maxillofacial surgery (average year of training 1.6 out of 5; average self-reported prior surgical experience 4.6 out of max. 10) was performed to evaluate the model’s effect on more experienced individuals. The training took place on two consecutive days and included basic microsurgical suturing exercises on the bench top and subsequent special suturing exercises on the chicken thigh and the head and neck RACE model. The first resident course day corresponded to course days 3 and 4 of the pregraduate microsurgical skills lab. The second day of the resident course included specific microsurgical exercises on the head and neck RACE model as they were performed on days 5 and 6 of the pregraduate course.

### Evaluation methods

The effect of implementing the RACE model on the learning curve of students after training on the conventional teaching model should be analyzed in detail and compared to standard learning curves in surgical education [27]. In addition, the evaluation was intended to provide information on the differences in learning success between pregraduate students and residents. For this purpose, each microsurgical performance parameter was assessed by an external rater on the basis of an established rating system [24, 28]. Performance levels were assessed prior and after the RACE model’s implementation into the course. Differences between the effects of the RACE models on the microsurgical performance of novice and more experienced course participants were evaluated by assessing a course for pregraduate students and a course for residents in maxillofacial surgery. In addition, self-assessment questionnaires were used to record the subjective perception of the models.

### Evaluation form – competency assessment tool

In collaboration with the Institute for Medical Teaching and Medical Education Research at the University of Würzburg, we developed an evaluation concept to assess the impact of the head and neck RACE model on the learning of microsurgical techniques. Its main tools are an evaluation form specifically developed and designed to assess microsurgical preparation and suturing skills, and a self-assessment questionnaire (Supplementary Material). We developed the assessment form (Supplementary Material Page 1) based on a published and validated competency assessment tool (CAT) [29, 30] and included parameters of surgical performance previously described by Muecke et al. and Scholz et al. [25, 26] to make the questionnaire applicable in microsurgical evaluation. Finally, adjustments were made by our own working group to reflect the educational characteristics of both the conventional chicken and the RACE head and neck model. The parameters of surgical performance and the items assessed in the self-assessment are listed as questions (Q) in the following table (Table 1). During each day of the course, questions Q1.1 to 4.2 of the CAT were assessed by an assessor using discrete verbal descriptor scales (Supplementary Material Page 1). At the end of the course day, the same rater summarized thematic groups of questions on the CAT for each student using graphical rating scales (Supplementary Material Page 2).

**Table 1:** Questions and parameters for the microsurgical CAT and the self-assessment questionnaire. The rater assessed Q1.1 to 4.2 based on discrete verbal descriptor scales. In Q5.1 to 5.3 the rater summarized thematic groups of the previous questions using graphical rating scales. Course participants answered a self-assessment containing Q6.1 to Q6.3 on the conventional chicken model and Q7.1 to 7.3 on the RACE model. The scale width of each parameter is shown in brackets. Higher scores represent better results

Question	Rating by the assessor on a verbal descriptor scale	Question	Rating by the assessor on a graphical rating scale
Q1.1	Vascular dissection (1–5)	Q5.1	Grouping Q1.1 to Q1.3 on a graphical rating scale: Preparation and tissue handling (0-100)
Q1.2	Tissue preserving technique (1–6)		
Q1.3	Distance of suture to vessel’s margin (1–5)		
Q2.1	Needle holder and forceps handling (1–6)	Q5.2	Grouping Q2.1 to Q2.3 on a graphical rating scale: Instrument and needle handling (0-100)
Q2.2	Needle handling (1–5)		
Q2.3	Thread handling/knot tying (1–4)		
Q3.1	Seam quality reverse side (1–5)	Q5.3	Grouping Q3.1 to Q4.2 on a graphical rating scale: Overall efficiency (0-100)
Q4.1	Time needed for procedure (1–6)		
Q4.2	Perfusion test (1–4)		
	**Self-assessment on a graphical rating scale: conventional chicken model**		
Q6.1	Tissue resemblance (0-100)		
Q6.2	Realistic spatial-anatomical conditions (0-100)		
Q6.3	Overall realism (0-100)		
	**Self-assessment on a graphical rating scale: RACE model**		
Q7.1	Tissue resemblance (0-100)		
Q7.2	Realistic spatial-anatomical conditions (0-100)		
Q7.3	Overall realism (0-100)		

By adjusting the scale width of the discrete verbal descriptors for each parameter, the microsurgical CAT does justice to the different degrees of difficulty of the various parameters [24] (S1). As a result of the inherently individual subjectivity of assessment criteria applied in a discrete verbal descriptor scale with behaviorally anchored rating scales, the working group opted for a single person assessment procedure.

### Self-assessment questionnaire and basic sewing test

A self-assessment questionnaire on tissue resemblance (Q6.1 and 7.1, Table 1 and Supplementary Material Pages 3 and 4), realistic spatial-anatomical conditions (Q6.2 and 7.2, Table 1 and Supplementary Material Pages 3 and 4) and overall realism (Q6.3 and 7.3, Table 1 and Supplementary Material Pages 3 and 4) was applied to assess the trainees’ perception of the realism of the two models used (Q6 and Q7, Table 1 and Supplementary Material Pages 3 and 4). We conducted the self-assessment before and after the introduction of the RACE model for the head and neck to test its effect on the perception of both models. The evaluation, including assessor ratings and self-assessment questionnaire, was conducted on course days 3 to 6 after completion of basic microsurgical suturing skills for the pregraduate. For the residents, we evaluated both course days.

To assess basic sewing skills independently of the model and the previously assessed microsurgical performance parameters, a sewing test was carried out in which the pregraduate participants had to sew 5 individual buttons on a simple sterile glove model at the end of each course day. The time taken was used as an assessment parameter to record and visualize the improvement in basic sewing skills during the course. The test was not evaluated as part of the ANOVA but was used to graphically represent the students’ basal suturing skills.

### Statistics and guidelines

R version 4.2.2 in combination with RStudio 2023.03.0 (R and RStudio, 4.2.2 and 2023.03.0, Posit PBC, Boston, Massachusetts, US) was used for analysis. Sample size computation was conducted using Basic Functions for Power Analysis (pwr 0.1-2) between groups of different sample size. A two-way repeated measures ANOVA based on Welch statistic was computed to inspect differences between groups [31–34]. The approach has demonstrated its resilience in the face of violations of the normality assumption and its applicability to small sample sizes [35]. Plots were created using ggplot2 (ggplot2, 3.4.4, Open Source/MIT license, Cambridge, Massachusetts, US). When calculating power for different sample sizes in subgroups for a two-way repeated measures ANOVA and a standardized mean difference of 1.5 scale points as primary endpoint on the CAT, a sample size of *n* = 29 can be considered sufficient for an alpha-level of 0.05 and a power of 80% [36]. The significance level for all p-values is 0.05.

All procedures were performed in accordance with relevant guidelines and regulations. All experimental protocols including the clinical patient scan were approved by the Ethics Committee of the Friedrich-Alexander University Erlangen-Nuremberg, which stated that no separate ethics application was necessary. As stated in the previous publication on the development of the RACE model, informed consent for online open-access publication was obtained from the scanned individual [8]. Informed consent for open access publication has been obtained from all course participants visible in Fig. 1. In addition, informed consent was obtained from all subjects and/or their legal guardian(s) for participation.

## Results

### Results of the microsurgical performance test

#### Assessor rating: microsurgical CAT

**Fig. 2:**
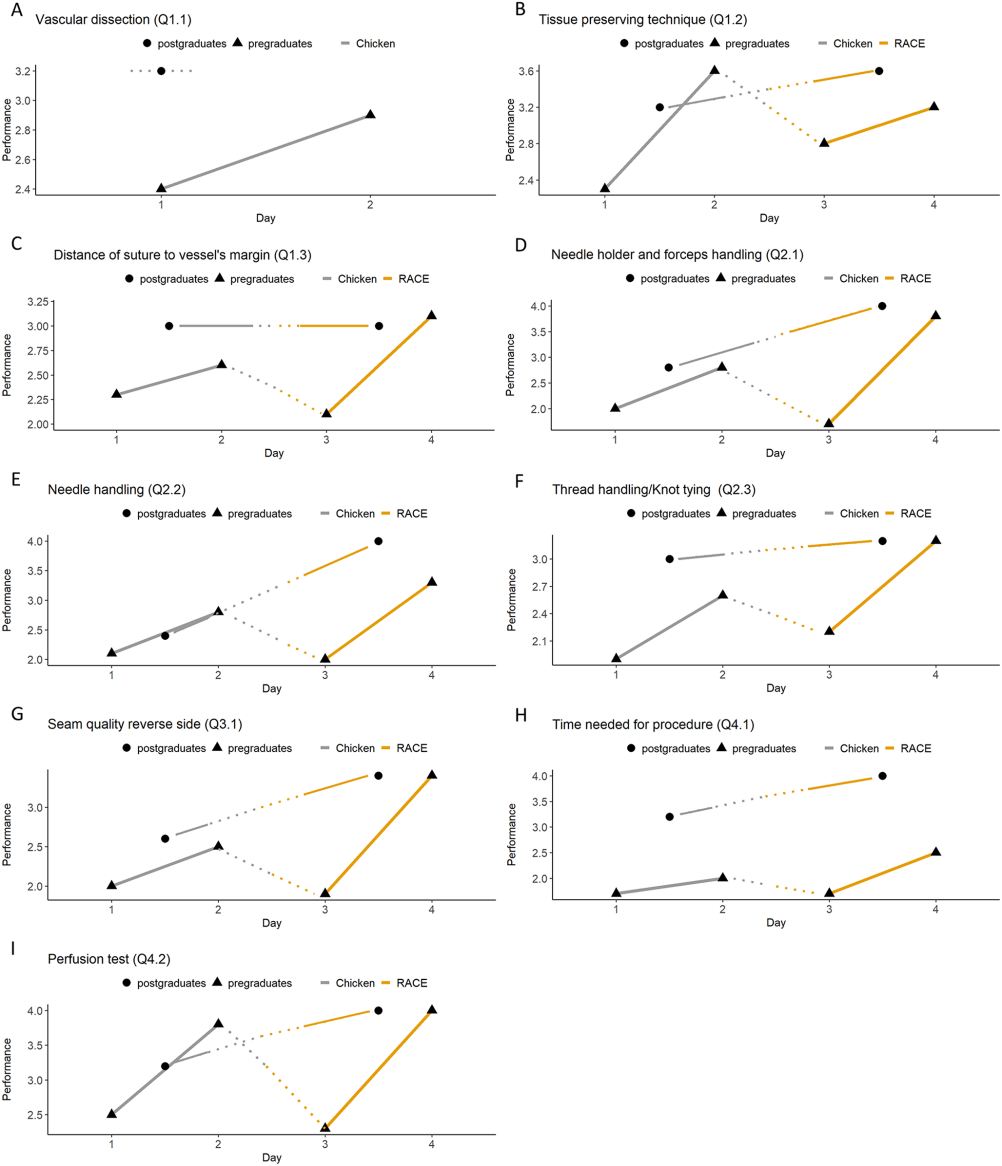
Statistical analysis based on the microsurgical competency assessment tool: Parameters of surgical performance. Two-Way ANOVA for repeated measures. The corresponding p-values are presented in Supplementary Material 2. **(a)** Vascular dissection (Q1.1). Y-axis shows the trainees’ performance from 1 to 5. The axis is scaled to the relevant range for better understanding. X-axis shows the day of the course from 1 to 2, as vascular dissection is only possible with the chicken model. **(b)** Tissue preserving technique (Q1.2). Y-axis shows the trainees’ performance from 1 to 6. The axis is scaled to the relevant range for better understanding. X-axis shows the day of the course from 1 to 4. **(c)** Distance of suture to vessels margin (Q1.3). Y-axis shows the trainees’ performance from 1 to 5. The axis is scaled to the relevant range for better understanding. X-axis shows the day of the course from 1 to 4. **(d)** Needle holder and forceps handling (Q2.1). Y-axis shows the trainees’ performance from 1 to 6. The axis is scaled to the relevant range for better understanding. X-axis shows the day of the course from 1 to 4. **(e)** Needle handling (Q2.2). Y-axis shows the trainees’ performance from 1 to 5. The axis is scaled to the relevant range for better understanding. X-axis shows the day of the course from 1 to 4. **(f)** Thread handling/Knot tying (Q2.3). Y-axis shows the trainees’ performance from 1 to 4. The axis is scaled to the relevant range for better understanding. X-axis shows the day of the course from 1 to 4. **(g)** Seam quality reverse side (Q3.1). Y-axis shows the trainees’ performance from 1 to 5. The axis is scaled to the relevant range for better understanding. X-axis shows the day of the course from 1 to 4. **(h)** Time needed for procedure (Q4.1). Y-axis shows the trainees’ performance from 1 to 6. The axis is scaled to the relevant range for better understanding. X-axis shows the day of the course from 1 to 4. **(i)** Perfusion test (Q4.2). Y-axis shows the trainees’ performance from 1 to 4. The axis is scaled to the relevant range for better understanding. X-axis shows the day of the course from 1 to 4

Following training on the conventional chicken models, the application of the head and neck RACE model resulted in an immediate deterioration in items Q1.2 to Q4.2 of the pregraduate course. With the exception of the tissue-preserving technique (Q1.2), the students’ performance in all items significantly improved on the second RACE model course day (Q1.2 *p* = 0.373, Q1.3 *p* = 0.003, Q1.4 *p* < 0.001, Q2.1 *p* = 0.022, Q2.2 *p* = 0.008, Q2.3 *p* = 0.014, Q3.1 *p* = 0.036, Q4.1 *p* = 0.036 and Q4.2 *p* = 0.002) and exceeded the values previously achieved on the conventional chicken model (Fig. 2; Tables 2 and 3). As vessel dissection (Q1.1) was not applicable to the RACE model, values are not presented for comparison between the respective models.

**Table 2:** Mean values, standard deviation and significance level for Q1.1 to Q7.3. Means and standard deviations are presented for each day of the course and for each model. Significance level is added for two-way repeated measures ANOVA. The p-values for the difference between two columns are shown in the second column. Significance level for all p-values is 0.05. The microsurgical CAT and self-assessment questionnaires are included. Data are provided for both pregraduate and postgraduate participants. As vascular dissection (Q1.1) is not applicable to the RACE model, no scores are presented. As the RACE model was introduced on days 3 and 2 of the course, no data are shown for the self-assessment for the previous days. A higher value indicates a superior rating

Question	Mean and Standard Deviation (in brackets) with *p*-values					
Rating by the assessor on a verbal descriptor scale						
	Pregraduates				Postgraduates	
	**Chicken model**		No vascular dissection on the RACE model		**Chicken model**	No vascular dissection on the RACE model
	**Day 1**	**Day 2**			**Day 1**	
Q1.1 Vascular dissection (1–5)	2.67 (1.18)	2.90 (1.20) *p* = 0.3629			3.20 (1.10)	
	**Pregraduates**				**Postgraduates**	
	**Chicken model**		**RACE model**		**Chicken model**	**RACE model**
	**Day 1**	**Day 2**	**Day 3**	**Day 4**	**Day 1**	**Day 2**
Q1.2 Tissue preserving technique (1–6)	2.30 (0.95)	3.60 (0.84) *p* = 0.006	2.80 (1.10)	3.20 (1.10) *p* = 0.373	3.20 (1.10)	3.60 (0.89) *p* = 0.545
Q1.3 Distance of suture to vessel’s margin (1–5)	2.30 (0.48)	2.60 (0.52) *p* = 0.193	2.10 (0.74)	3.10 (1.10) *p* = 0.003	3.00 (0.00)	3.00 (0.00) *p* = 1.00
Q2.1 Needle holder and forceps handling (1–6)	2.00 (0.82)	2.80 (1.32) *p* = 0.086	1.70 (1.25)	3.80 (1.14) *p* < 0.001	2.80 (1.10)	4.00 (0.00) *p* = 0.070
Q2.2 Needle handling (1–5)	2.10 (0.74)	2.80 (1.03) *p* = 0.088	2.00 (1.15)	3.30 (1.16) *p* = 0.022	2.40 (0.89)	4.00 (0.00) *p* = 0.016
Q2.3 Thread handling/knot tying (1–4)	1.90 (0.74)	2.60 (0.70) *p* = 0.132	2.20 (0.92)	3.20 (0.42) *p* = 0.008	3.00 (0.00)	3.20 (0.45) *p* = 0.373
Q3.1 Seam quality reverse side (1–5)	2.00 (0.94)	2.50 (0.53) *p* = 0.244	1.90 (0.88)	3.40 (1.17) *p* = 0.014	2.60 (0.55)	3.40 (0.89) *p* = 0.134
Q4.1 Time needed for procedure (1–6)	1.70 (1.16)	2.00 (1.33) *p* = 0.193	1.70 (1.16)	2.50 (1.08) *p* = 0.036	3.20 (0.45)	4.00 (0.00) *p* = 0.016
Q4.2 Perfusion test (1–4)	2.50 (1.35)	3.80 (0.42) *p* = 0.013	2.30 (1.25)	4.00 (0.00) *p* = 0.002	3.20 (1.30)	4.00 (0.00) *p* = 0.242
**Rating by the assessor on a graphical rating scale**						
Q5.1 Preparation and tissue handling (0-100)	45.00 (24.29)	48.33 (11.69) *p* = 0.777	42.50 (5.00)	42.50 (18.93) *p* = 1.00	44.00 (19.81)	60.00 (17.32) *p* = 0.211
Q5.2 Instrument and needle handling (0-100)	48.00 (26.06)	53.00 (21.50) *p* = 0.212	45.00 (20.68)	52.00 (26.06) *p* = 0.404	47.00 (17.89)	64.00 (18.17) *p* = 0.174
Q5.3 Overall Efficiency (0-100)	37.00 (24.06)	51.50 (20.01) *p* = 0.045	44.00 (27.57)	44.00 (23.19) *p* = 1.00	43.00 (21.10)	75.00 (7.07) *p* = 0.024
**Self-assessment on a graphical rating scale: Conventional chicken model**						
Q6.1 Tissue resemblance (0-100)	90.67 (6.16)	88.13 (5.67) *p* = 0.650	83.89 (9.93)	84.44 (5.27) *p* = 0.891	84.00 (8.94)	84.00 (8.94) *p* = 1.00
Q6.2 Realistic spatial-anatomical conditions (0-100)	57.00 (25.76)	54.50 (22.19) *p* = 0.863	54.44 (21.86)	52.22 (19.86) *p* = 0.707	68.00 (19.24)	42.00 (17.89) *p* = 0.057
Q6.3 Overall realism (0-100)	73.00 (16.56)	72.25 (18.26) *p* = 0.899	71.11 (12.94)	70.00 (11.99) *p* = 0.824	80.00 (10.00)	62.00 (14.83) *p* = 0.059
**Self-assessment on a graphical rating scale: RACE model**						
Q7.1 Tissue resemblance (0-100)			39.17 (24.17)	51.67 (23.17) *p* = 0.416		60.00 (12.25)
Q7.2 Realistic spatial-anatomical conditions (0-100)			88.33 (10.33)	90.00 (6.32) *p* = 0.787		91.60 (2.30)
Q7.3 Overall realism (0-100)			68.33 (14.72)	76.67 (10.33) *p* = 0.383		74.00 (15.17)

**Table 3:** Significance levels for the direct comparison between the courses and models as well as the specific course days. Comparison between the conventional chicken model and the RACE model for head and neck as well as between pregraduates and postgraduates and the different course days with the respective p-values. P-values below 0.001 are simplified as < 0.001. The significance level for all p-values is 0.05

Focus	Chicken model		RACE model		Model		Pregraduates Chicken model		Pregraduates RACE model		Pregraduates		Postgraduates	
Comparison	pregraduate	postgraduate	pregraduate	postgraduate	Chicken model	RACE model	Day 1	Day 2	Day 3	Day 4	Chicken model	RACE model	Chicken model	RACE model
Q1.1	**0.041**		not applicable		not applicable		0.3629		not applicable		not applicable		not applicable	
Q1.2	0.663		0.277		0.56		**0.006**		0.373		0.905		0.545	
Q1.3	**< 0.001**		0.103		0.579		0.193		**0.003**		0.569		1.00	
Q2.1	0.494		**0.002**		0.052		0.086		**< 0.001**		0.428		0.070	
Q2.2	0.915		**< 0.001**		0.136		0.088		**0.022**		0.583		**0.016**	
Q2.3	**< 0.001**		0.096		0.079		0.132		**0.008**		0.093		0.373	
Q3.1	0.276		0.162		0.070		0.244		**0.014**		0.239		0.134	
Q4.1	**< 0.001**		**< 0.001**		0.319		0.193		**0.036**		0.512		**0.016**	
Q4.2	0.940		**0.005**		0.628		**0.013**		**0.002**		1.00		0.242	
Q5.1	0.803		0.093		0.602		0.777		1.00		0.556		0.211	
Q5.2	0.723		0.148		0.778		0.212		0.404		0.787		0.174	
Q5.3	0.910		**< 0.001**		0.363		**0.045**		1.00		0.973		**0.024**	
Q5.4	0.181		**< 0.001**		0.207		0.393		0.494		0.915		**< 0.001**	
Q6.1	0.253		0.971		0.067		0.650		0.891		**0.028**		1.00	
Q6.2	0.272		0.262		0.233		0.863		0.707		0.739		0.057	
Q6.3	0.249		0.284		0.196		0.899		0.824		0.677		0.059	
Q7.1	not applicable		0.116		0.500		0.483		0.416		0.764		not applicable	
Q7.2	not applicable		0.362		0.414		0.191		0.787		0.358		not applicable	
Q7.3	not applicable		0.852		0.517		0.454		0.383		0.560		not applicable	

In the training of postgraduates, improvement was observed for all items when switching to the RACE model (Q2.2 and Q4.1 showed significant improvement with *p* = 0.016 and *p* = 0.016 respectively), with the exception of distance of suture to vessel’s margin (Q1.3), which remained unchanged. The vascular dissection (Q1.1) was excluded from the comparison for all trainees, as it was not assessable on the head and neck RACE model. The complete set of values can be found in Figure, Tables 2 and 3.

#### Assessor rating: graphic rating scale

**Fig. 3:**
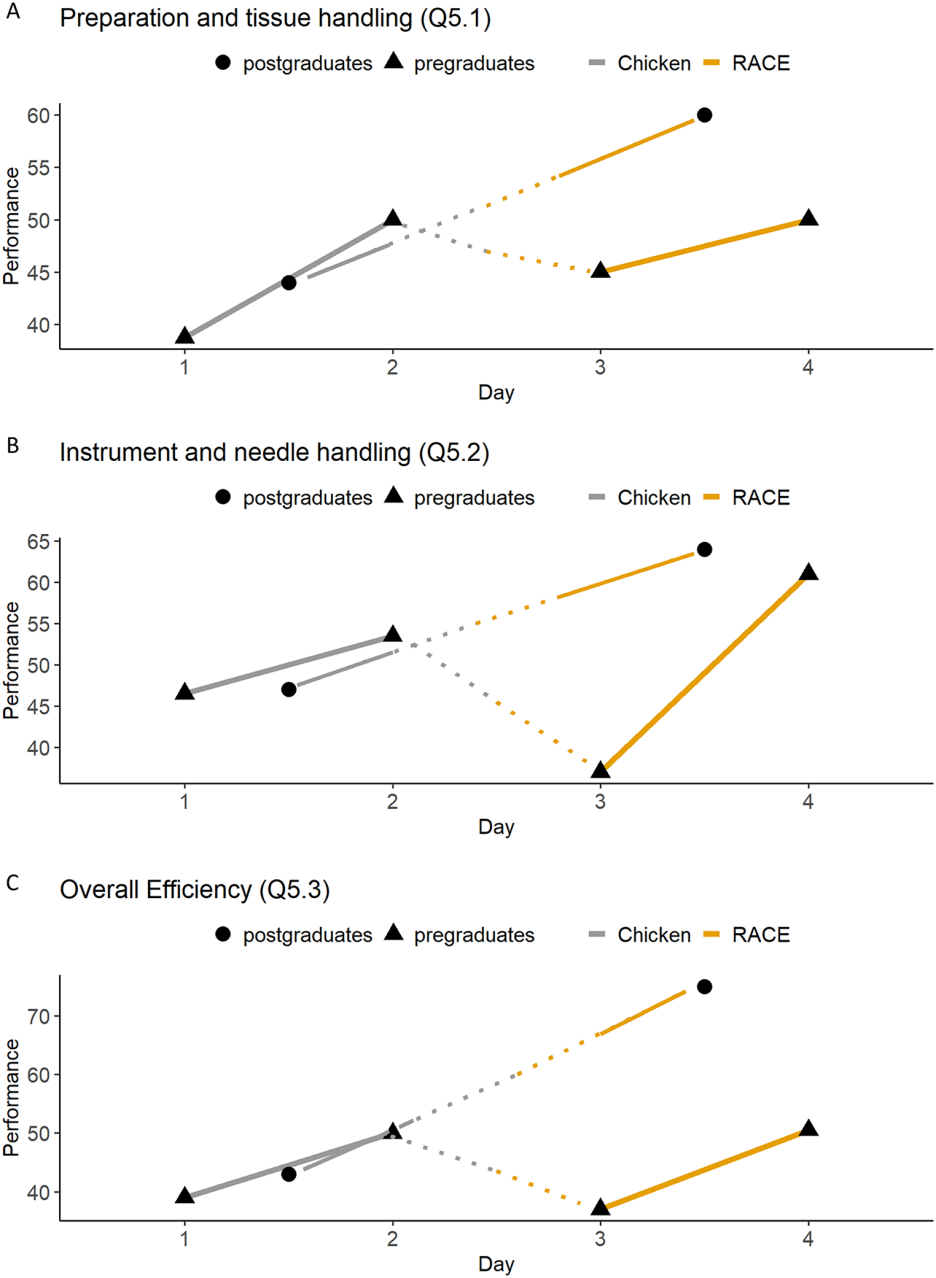
Statistical analysis based on the microsurgical competency assessment tool: Summarization of the parameters of surgical performance by use of graphical rating scales. Paired samples t-test and Two-Way ANOVA. The corresponding p-values are presented in Supplementary Material 2. **(a)** Preparation and tissue handling (Q5.1). Y-axis shows the trainees’ performance from 0 to 100. The axis is scaled to the relevant range for better understanding. Y-axis shows the day of the course from 1 to 4. **(b)** Instrument and needle handling (Q5.2). Y-axis shows the trainees’ performance from 0 to 100. The axis is scaled to the relevant range for better understanding. X-axis shows the day of the course from 1 to 4. **(c)** Overall Efficiency (Q5.3). Y-axis shows the trainees’ performance from 0 to 100. The axis is scaled to the relevant range for better understanding. X-axis shows the day of the course from 1 to 4

A graphical representation of the previously described assessors’ evaluation from the microsurgical CAT, presented in three groups, demonstrates that all three graphs (Q5.1 to 5.3) exhibit an immediate regression when transitioning from the conventional chicken model to the head and neck RACE model in the pregraduate group (Fig. 3A-C; Tables 2 and 3 and Supplementary Material Page 2). However, after an additional day of practice, all graphs returned to or exceeded the previous level of the chicken model (Q5.1 *p* = 1.00, Q5.2 *p* = 0.404, Q5.3 *p* = 1.00; Fig. 3A-C; Tables 2 and 3). For residents, all three items (Q5.1 *p* = 0.211, Q5.2 *p* = 0.174 and Q5.3 *p* = 0.024 ) demonstrated superior and significantly superior performance compared to the conventional chicken model, once the transition to the RACE model for head and neck has been implemented (Fig. 3A-C; Tables 2 and 3). No initial decline in performance was observed. The complete set of values can be found in Fig. 3A-C; Tables 2 and 3.

#### Assessor rating: sewing test for pregraduates

**Fig. 4:**
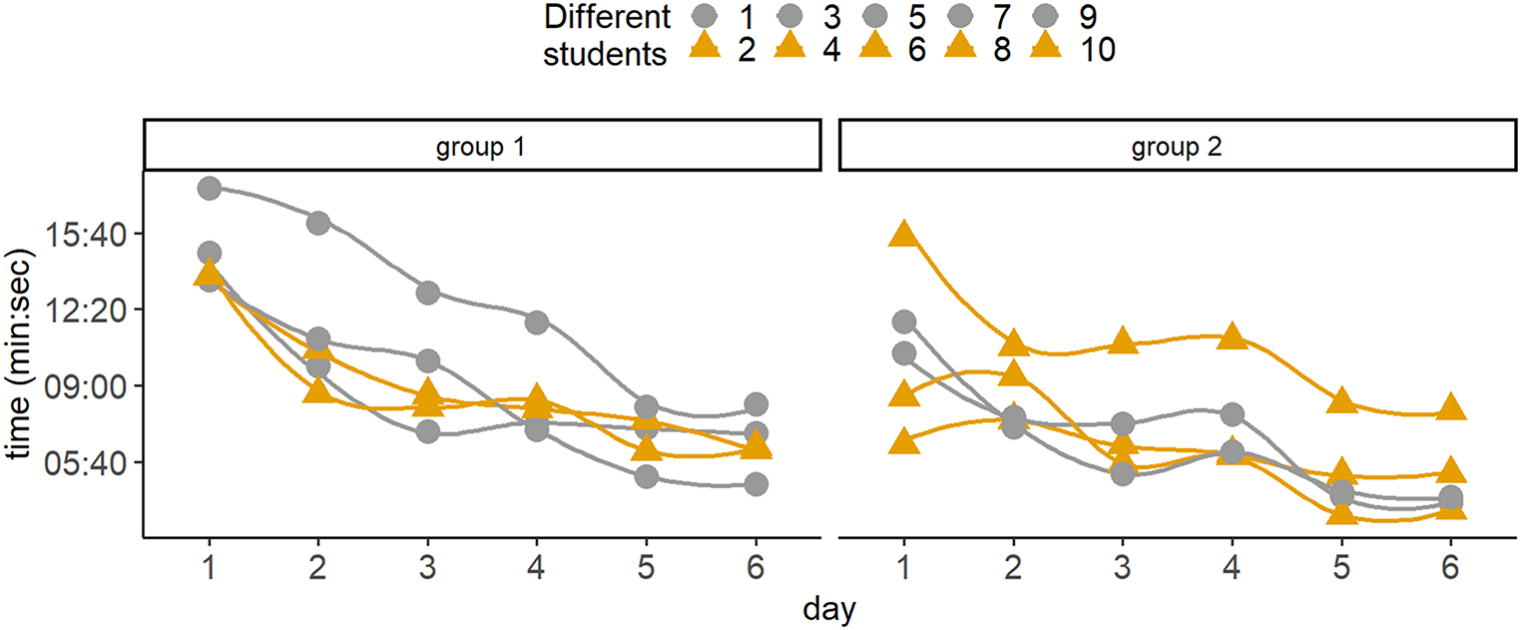
Test for the assessment of basal sewing skills on the glove model. Time chart of pregraduate performance in the sewing test on a simple sterile glove model at the end of each course day. Y-axis shows time (min: sec). X-axis shows pregraduate course days 1 to 6 for group 1 and 2 respectively. Both scales are linear. Comparing time for day 5 and day 6 irrespectively of group, students require significantly less time on day 5 and day 6 (M = 5.55, SD = 1.67) than on average on day 1 to day 4 (M = 9.46, SD = 3.17), *p* < 0.001

The results for the suture test in 10 pregraduates (5 per group), over the course of the training program to assess the time taken to place 5 single button welds are presented in the form of a time graph. For both courses, the time decreased steadily from a maximum of 14:43 min: seconds on day 1 for group 1 to a minimum of 4:55 min: seconds on day six for group 2 (Fig. 4). Following a period of stagnation from day 3 to day 4, an increase in performance was observed from day 4 to day 5 with the introduction of the head and neck RACE model to the course. When comparing time for day 5 and day 6, students require significantly less time on day 5 and day 6 (median (M) = 5.55, standard deviation (SD) = 1.67) than on average on day 1 to day 4 (M = 9.46, SD = 3.17), *p* < 0.001 (Fig. 4).

## Results of the self-assessment

### Self-assessment on the realism of the two models in comparison

The questionnaires pertaining to the chicken model were completed at the conclusion of each course day. Furthermore, the questionnaires associated with the head and neck RACE model were answered at the end of each course day, once its implementation had been completed.

**Fig. 5:**
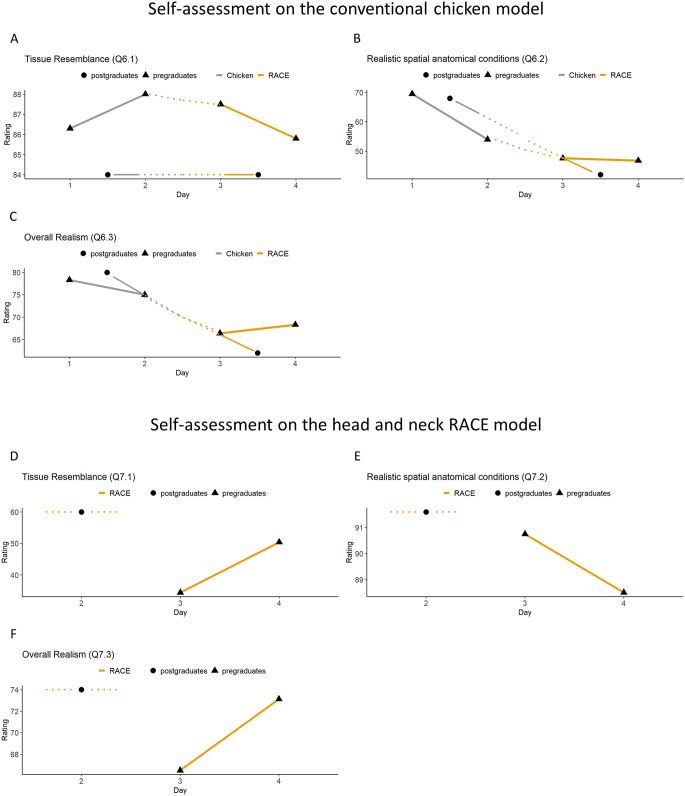
Statistical analysis based on the self-assessment questionnaire: Assessment of the chicken model **(A-C)** and the head and neck RACE model **(D-F)**. Paired samples t-test and Two-Way ANOVA. The corresponding p-values are presented in Supplementary Material 2. **(a)** Tissue Resemblance (Q6.1). Y-axis shows the trainees’ self-report from 0 to 100. The axis is scaled to the relevant range for better understanding. X-axis shows the day of the course from 1 to 4. **(b)** Realistic spatial-anatomical conditions (Q6.2) Y-axis shows the trainees’ self-report from 0 to 100. The axis is scaled to the relevant range for better understanding. X-axis shows the day of the course from 1 to 4. **(c)** Overall Realism Q6.3. Y-axis shows the trainees’ self-report from 0 to 100. The axis is scaled to the relevant range for better understanding. X-axis shows the day of the course from 1 to 4. **(d)** Tissue Resemblance (Q7.1). Y-axis shows the trainees’ self-report from 0 to 100. The axis is scaled to the relevant range for better understanding. X-axis shows the day of the course from 2 to 4 as a self-report on the RACE model was only possible on day 3 and 4 for the pregraduates and day 2 for the postgraduates. **(e)** Realistic spatial-anatomical conditions (Q7.2). Y-axis shows the trainees’ self-report from 0 to 100. The axis is scaled to the relevant range for better understanding. X-axis shows the day of the course from 2 to 4 as a self-report on the RACE model was only possible on day 3 and 4 for the pregraduates and day 2 for the postgraduates. **(f)** Overall Realism (Q7.3). Y-axis shows the trainees’ self-report from 0 to 100. The axis is scaled to the relevant range for better understanding. X-axis shows the day of the course from 2 to 4 as a self-report on the RACE model was only possible on day 3 and 4 for the pregraduates and day 2 for the postgraduates

Questions Q6.1 to Q6.3 describe the trainees’ opinion of the conventional chicken model before and after the introduction of the RACE model. The scores for tissue resemblance remain relatively stable throughout the course (Q6.1 *p* = 0.650 and *p* = 0.891, Fig. 5A; Tables 2 and 3). Before (M = 65.2, SD = 16.8) the introduction of the RACE model, pregraduates rated the chicken model significantly higher than after (M = 46.2, SD = 21.3) the introduction of the RACE model *p* = 0.028. For realistic spatial-anatomical conditions and overall realism, both groups rated the chicken model higher prior to the introduction of the RACE model (Q6.2 *p* = 0.739 and 0.057, Q6.3 *p* = 0.677 and *p* = 0.059, Fig. 5B and C; Tables 2 and 3).

Items Q7.1 to 7.3 describe the trainees’ opinion of the RACE model after its application on days 3 and 4 for the pregraduates and on day 2 for the postgraduates. There was no significant difference in tissue resemblance (Q7.1 *p* = 0.116, Fig. 5D; Tables 2 and 3) between the assessment of the RACE model by pregraduates and postgraduates. However, postgraduates rated the model higher (M = 60, SD = 12.2) than pregraduates (M = 42.4, SD = 23.3) (Fig. 5D; Tables 2 and 3). For realistic spatial-anatomical conditions, there was no significant difference between pre- and postgraduate participants, with all values at a generally high level in the upper fifth of the scale (Q7.2 *p* = 0.362, Fig. 5E; Tables 2 and 3). The postgraduates rated the model slightly higher (M = 91.6, SD = 2.30) than the pregraduates (M = 89.6, SD = 7.17). Regarding overall realism there was no significant difference between pre- and postgraduates. Again, postgraduates rated the model slightly higher (M = 74, SD = 15.2) than pregraduates (M = 69.8, SD = 12.9) (Q7.3 *p* = 0.852, Fig. 5F; Tables 2 and 3).

Detailed results are presented in the form of Figs. 2, 3, 4 and 5; Tables 1, 2 and 3. The microsurgical CAT as well as the self-assessment questionnaires are added as Supplementary Material Page 1–5.

## Discussion

Microsurgical experience and the surgeon’s training have previously been identified as the main factors contributing to the success of vascular microanastomosis and microvascular tissue transfer [9–11]. Cristiancho et al. took an interesting approach by focusing primarily on the experienced surgeon’s ability to respond to unfamiliar intraoperative environments and events as one of the unique aspects of surgery [37]. Non-ergonomic and therefore challenging operating conditions are not or inadequately represented in current training models. We have identified this discrepancy between reality and the teaching environment as a major research gap in microsurgical training and have focused on possible solutions. As a consequence, we took a new approach with the development of the head and neck RACE model using modern 3D scanning and printing technology to transfer the intraoperative environment as accurately as possible into the training environment and thus enable the trainee to come into contact with the special challenges of head and neck microsurgery, which otherwise only occur during real surgery [8]. Furthermore, unlike current live or cadaveric animal models, the RACE model provides a reusable and therefore future-oriented training option [38].

Evaluation data show that the microsurgical performance of pregraduate students significantly deteriorates when switching to the head and neck RACE model (Figs. 2B-I and 3A-C). This indicates a successful simulation of realistic and therefore more challenging surgical conditions compared to the conventional chicken model. Contrary to the pregraduate course and in line with the ability of a more experienced surgeon to cope with unexpected intraoperative conditions as described by Christiancho et al., the implementation of the RACE model in the postgraduate/resident course did not show a decrease in performance when considering the microsurgical performance parameters (Figs. 2B-I and 3A-C). We therefore conclude that general surgical experience also has a positive effect on the learning of microsurgical skills. A detailed analysis of the microsurgical performance parameters allows further conclusions to be drawn. In contrast to instrument, needle and suture handling (Fig. 2C-G), which in microsurgery only differs from conventional surgery in that it requires less space and smaller instruments and needles, the tissue-preserving technique presents previously unknown challenges. One such challenge is preserving the adventitia to avoid thrombus formation. We observed that tissue preserving technique (Fig. 2B) shows a significantly greater initial performance increase in pregraduates than the other parameters surveyed. This suggests a lack of instruction in tissue preparation and general tissue handling in the pregraduate curriculum prior to this course, resulting in a steeper initial learning curve (Fig. 2B). Regarding the other parameters of surgical performance for pregraduate students, the implementation of the RACE model on day 3 had a pronounced influence as we observed a stronger initial drop followed by a stronger subsequent increase in performance (Fig. 2C-G) in comparison to the previously mentioned tissue preserving technique (Fig. 2B). We conclude that the handling of instruments and needle and thread is much more influenced by the available space and the resulting ergonomics than the handling of tissue.

Current educational research shows that struggling is absolutely critical to mastery [39]. The previously described drop in performance of pregraduate students when switching from a conventional model to a practice environment with realistic spatial-anatomical conditions on the RACE model (Fig. 2B-I) can be regarded as an initial struggle, which is then followed by an immediate and even stronger increase in performance than on the conventional chicken model (Fig. 2C-I). Usually, classical learning curves in surgery show negative exponential behavior with an initially rapid and then plateauing learning increase [27]. It is noteworthy that the learning curve observed in the pre-graduate course challenges these expectations. Following an initial decline with the introduction of the RACE model, we observed a more pronounced increase in learning than previously observed with the conventional model (Fig. 2C-I). In addition, the basic suturing skills of the pregraduate students, measured by the time required for 5 sutures under free space conditions on the glove model, shows a visible and significant improvement in the courses transitioning from the conventional chicken model to the RACE model (day 4 to day 5, Fig. 4). We therefore conclude that the introduction of the RACE model into microsurgical training improves the overall microsurgical learning process of medical students, despite or due to initial difficulties and deterioration.

Surgical education profits from high-fidelity simulation as it implies greater learner engagement [20, 22, 23, 40]. With the development of the head and neck RACE model, we aimed to improve microsurgical training by creating a high-fidelity learning environment through the implementation of realistic spatial-anatomical conditions as well as a proper tissue resemblance. So far, and even considering that we have used flexible resin, the complexity of real tissue cannot be fully replicated in a standard 3D printed model. It is therefore particularly interesting that similar values for tissue resemblance were achieved for the RACE model as for the conventional chicken model (Fig. 5D and F). This indicates that the training situation is assessed very realistically overall and that this type of resin can therefore be recommended for future RACE models. We consider the main objective of developing a realistic high-fidelity model while implementing realistic spatial-anatomical conditions to be successful, based on the average rating of 91.1 out of 100 points for both pregraduates and residents (Fig. 5E). Remarkably, in the self-assessment all three parameters (tissue resemblance, realistic spatial-anatomical conditions and overall realism) show a poorer assessment of the conventional model after familiarization with the RACE model.

As part of the evaluation of the microsurgery course and the associated establishment of the RACE model, the newly developed evaluation form, which was based on current literature [10, 24, 26, 29, 30] and modified by our working group according to microsurgical standards, proved to be practicable. We can therefore recommend the evaluation form (CAT and self-assessment questionnaires) enclosed as Supplementary Material for future microsurgical evaluations and the investigation of new teaching models.

One of the main limiting factors of the study is the total number of individuals observed (15). Larger studies need to be conducted to obtain more in-depth data on the learning behavior of pregraduate students and residents in microsurgical training using the head and neck RACE model. As there are discrepancies in the subjective perception of trainees’ microsurgical skills between different assessors, the working group decided in favor of a single assessor approach. To address this limitation, it would be interesting to match the 3D-printed training model with recently introduced features such as smartphone applications and artificial intelligence in order to improve knowledge about the reliability of RACE models in daily clinical practice [41, 42]. Although some microvascular surgeons perform trimming of the adventitia and others do not, the procedure should at least be taught in microsurgical training. Therefore, the non-applicability of vessel dissection should be stated as a limitation of the head and neck RACE model in use. However, the use of recently developed triple-layered synthetic vessels for microsurgery training may address the current limitation in the future, as any synthetic or organic vessel can be easily applied to the head and neck RACE model [43].

In summary, we were able to confirm a successful simulation and transfer of the complex and challenging microsurgical conditions in the head and neck region trough application of the head and neck RACE model in a detailed evaluation. This evaluation demonstrated feasibility of the head and neck RACE model and now paves way for the development of further surgical and general medical RACE models based on the previously published step-by-step RACE workflow [8].

## Conclusions

The head and neck RACE model offers a challenging and practical addition to microsurgery teaching. Applying the models to pregraduate students results in initial deterioration of microsurgical practice followed by a steeper learning curve compared to learning on conventional teaching models as well as the expected standard learning curve. In contrast, we observed no initial deterioration in residents, which suggests a more experienced handling of unfamiliar surgical settings and a faster ability to adapt. The head and neck RACE model and the newly developed and applied evaluation form proved to be feasible in microsurgical training. We can therefore recommend the development of further RACE models in other specialties based on the RACE workflow.

## Electronic supplementary material

Below is the link to the electronic supplementary material.

Supplementary Material 1

Supplementary Material 2

## Data Availability

No datasets were generated or analysed during the current study.

## Abbreviations

RACE model: Realistic anatomical condition experience model
CAT: Competency Assessment Tool
OSCE: Objective structured clinical examination
VAS: Visual analogue scale
M: Mean
SD: Standard deviation
p: Significance level
