# Supplementary material for: Evaluation of a 3D printed training model with realistic spatial-anatomical conditions for head and neck microsurgery
Source: Clin Oral Investig. 2025 Apr 8;29(5):228. doi: 10.1007/s00784-025-06314-4 (PMC11978532; doi:10.1007/s00784-025-06314-4)
Supplement: Supplementary file 1 — Supplementary Material 1 [file 784_2025_6314_MOESM1_ESM.pdf]

Competency Assessment Tool (CAT) for microsurgical training model and programme

Trainee name: ______________________________ **Assessor:** ______________________ **Date:** ____/____/____

1. Please √ the description that best describes the observed performance:

TASK	DISSECTION AND TISSUE HANDLING			INSTRUMENT USE			SEWING TECHNIQUE			END-PRODUCT QUALITY		
	**Vascular dissection (Q1.1)**			**Needle holder and forceps handling (Q2.1)**			**Seam quality reverse side (Q3.1)**			**Time needed for procedure (Q4.1)**		
	Damaging (1)	Tissue damaging technique, forceful		Uncoordinated (1)	Stiff and uncontrolled movements, overshooting		Poor (1)	No sufficient seam achieved on the rear side		Slow (1)	Performance slower than 45	
	Hesitant (2)	Controlled movements but hesitant and inefficient		Hesitant (2)	Controlled movements, but hesitant and inefficient		Sufficient (2)	Irregular suture, unsafe handling of the approximator		Hesitant (3)	Performance in under 35 min	
	Safe (4)	Safe and clean dissection technique		Skilful (4)	Smooth, controlled and meaningful movements		Good (3)	Slightly irregular, sufficient suture		Swift (4)	Performance in under 25 min	
	Versatile (5)	Efficient, economic and safe dissection		Versatile (6)	Masterful instrument use, effective movements		Excellent (5)	Front like back, sufficient		Rapid (6)	Performance in under 15 min	
	Comments:											
	**Tissue preserving technique (Q1.2)**			**Needle handling (Q2.2)**						**Perfusion test (Q4.2)**		
	Aggressive (1)	Forceful, damaging tissue handling, unnecessary endothelium damage		Uncoordinated (1)	Stiff and uncontrolled movements, overshooting, needle damaging					Insufficient (1)	Severe leakage, total perfusion obstruction	
	Inefficient (2)	Hesitant moves, slight tissue damage		Hesitant (2)	Controlled movements, but hesitant and inefficient					Moderately insufficient (2)	Moderate leakage, moderate perfusion obstruction	
	Safe (4)	Safe tissue handling, no essential tissue damage		Skilful (4)	Smooth, controlled and meaningful movements					Slightly insufficient (3)	Slight leakage, slight perfusion obstruction	
	Efficient (6)	Smooth and efficient tissue handling, perfect tissue preservation		Versatile (5)	Masterful needle use, effective movements					Sufficient (4)	No leakage, continuous	
	Comments:											
	**Distance of suture to vessel's margin (Q1.3)**			**Thread handling/ knot tying (Q2.3)**								
	Inaccurate (1)	Dilettante spacing, inconsistent, randomly		Hazardous (1)	Insufficient view, uncontrolled movements, thread damaging							
	Irregular (2)	Hesitant and irregular suturing, acceptable spacing		Laborious (2)	Awkward and repeated unnecessary attempts, insufficient knot technique							
	Skilful (3)	Right spacing, slightly irregular		Efficient (3)	Skilful thread handling, sufficient knot technique							
	Masterly (5)	Perfect und even suture placing on both margins		Versatile (4)	Highly efficient and skilful use of thread, safe knotting technique							
	Comments:											

© Manuel Olmos, Friedrich-Alexander-University Erlangen-Nürnberg

To be completed by the assessor:

Please **mark with an asterisk** the point that best represents the level of individual performance.		Comments
**PREPARATION AND TISSUE HANDLING** exposure of the vascular bundle, adventitia trimming if needed, overall tissue handling and preserving technique **(Q5.1)**	**Very Poor** **Pass** **Excellent**<br>0 10 20 30 40 50 60 70 80 90 100<br>Unable to accomplish goal, marked hesitation, uncontrolled movements, lack of procedure-specific and anatomical knowledge and poor technical skills.<br>Able to accomplish goal without hesitation, excellent progress and flow. Consistent demonstration of procedure-specific and anatomical knowledge. Skills adapted to the context of the procedure.	
**INSTRUMENT AND NEEDLE HANDLING** Safe, decisive and efficient instrument and needle usage **(Q5.2)**	**Very Poor** **Pass** **Excellent**<br>0 10 20 30 40 50 60 70 80 90 100<br>Unable to accomplish goal, marked hesitation, uncontrolled movements, lack of procedure-specific and anatomical knowledge and poor technical skills.<br>Able to accomplish goal without hesitation, excellent progress and flow. Consistent demonstration of procedure-specific and anatomical knowledge. Skills adapted to the context of the procedure.	
**OVERALL EFFICIENCY** Output per time **(Q5.3)**	**Very Poor** **Pass** **Excellent**<br>0 10 20 30 40 50 60 70 80 90 100<br>Unable to accomplish goal, marked hesitation, uncontrolled movements, lack of procedure-specific and anatomical knowledge and poor technical skills.<br>Able to accomplish goal without hesitation, excellent progress and flow. Consistent demonstration of procedure-specific and anatomical knowledge. Skills adapted to the context of the procedure.	

2. Please **circle** the description that best represents your level of agreement with the statement below:

This surgeon/trainee has demonstrated the knowledge and skill to undertake a supervised microanastomosis.

No ◄······► Borderline no ··· Undecided ··· Borderline yes ··· Yes

© Manuel Olmos, Friedrich-Alexander-University Erlangen-Nürnberg

To be completed by the trainee on the conventional chicken model:

Please **mark with an asterisk** the point that best represents the level of individual performance.	
**TISSUE RESEMBLANCE**<br>How well could the chicken model simulate the tissue properties of human arteries?<br>**(Q6.1)**	**Very Poor** **Pass** **Excellent**<br>0 10 20 30 40 50 60 70 80 90 100<br>Poor resemblance of tissue properties, crucial differences in consistency and handling<br>Perfect resemblance of tissue properties, crucial differences in consistency and handling
**REALISTIC SPATIAL-ANATOMICAL CONDITIONS**<br>How well could the chicken model simulate the spatial anatomical conditions?<br>**(Q6.2)**	**Very Poor** **Pass** **Excellent**<br>0 10 20 30 40 50 60 70 80 90 100<br>Poor resemblance of spatial anatomical conditions including no presence of undercutting areas and lack of space<br>Perfect resemblance of spatial anatomical conditions including the presence of undercutting areas and lack of space
**OVERALL REALISM**<br>General realism of the model<br>**(Q.6.3)**	**Very Poor** **Pass** **Excellent**<br>0 10 20 30 40 50 60 70 80 90 100<br>The model fails to simulate anatomical conditions and the surgical procedure of suturing a microanastomosis.<br>The model manages to simulate anatomical conditions and the surgical procedure when suturing a microanastomosis.

© Manuel Olmos, Friedrich-Alexander-University Erlangen-Nürnberg

To be completed by the trainee on the 3D printed RACE model:

Please **mark with an asterisk** the point that best represents the level of individual performance.	
**TISSUE RESEMBLANCE**<br>How well could the RACE model simulate the tissue properties of human arteries compared to the conventional model?<br>**(Q7.1)**	**Very Poor** **Pass** **Excellent**<br>0 10 20 30 40 50 60 70 80 90 100<br>Unable to accomplish goal, marked hesitation, uncontrolled movements, lack of procedure-specific and anatomical knowledge and poor technical skills.<br>Able to accomplish goal without hesitation, excellent progress and flow. Consistent demonstration of procedure-specific and anatomical knowledge. Skills adapted to the context of the procedure.
**REALISTIC SPATIAL ANATOMICAL CONDITIONS**<br>How well could the RACE model simulate the spatial anatomical conditions compared to the conventional model?<br>**(Q7.2)**	**Very Poor** **Pass** **Excellent**<br>0 10 20 30 40 50 60 70 80 90 100<br>Unable to accomplish goal, marked hesitation, uncontrolled movements, lack of procedure-specific and anatomical knowledge and poor technical skills.<br>Able to accomplish goal without hesitation, excellent progress and flow. Consistent demonstration of procedure-specific and anatomical knowledge. Skills adapted to the context of the procedure.
**OVERALL REALISM**<br>How would you rate the RACE model´s overall realism compared to the conventional model?<br>**(Q7.3)**	**Very Poor** **Pass** **Excellent**<br>0 10 20 30 40 50 60 70 80 90 100<br>Unable to accomplish goal, marked hesitation, uncontrolled movements, lack of procedure-specific and anatomical knowledge and poor technical skills.<br>Able to accomplish goal without hesitation, excellent progress and flow. Consistent demonstration of procedure-specific and anatomical knowledge. Skills adapted to the context of the procedure.

Manuel Olmos, Joy Backhaus, Rainer Lutz MD, DMD, Christopher-Phillip Nobis MD, DMD, Sarah Koenig MD, Marco Kesting MD, DMD, Manuel Weber MD, DMD

Evaluation of a 3D printed training model with realistic spatial-anatomical conditions for head and neck microsurgery

Clinical Oral Investigations

Address Correspondence and request for reprints to:

Manuel Weber MD, DMD

Department of Oral and Cranio-Maxillofacial Surgery

Friedrich-Alexander University Erlangen-Nürnberg

Glueckstrasse 11, 91054 Erlangen, Germany

Phone: +49-9131-85-43749

Email: manuel.weber@uk-erlangen.de

© Manuel Olmos, Friedrich-Alexander-University Erlangen-Nürnberg

© Manuel Olmos, Friedrich-Alexander-University Erlangen-Nürnberg
