# Supplementary material for: Evaluation of a 3D printed training model with realistic spatial-anatomical conditions for head and neck microsurgery
Source: Clin Oral Investig. 2025 Apr 8;29(5):228. doi: 10.1007/s00784-025-06314-4 (PMC11978532; doi:10.1007/s00784-025-06314-4)
Supplement: Supplementary file 2 — Supplementary Material 2 [file 784_2025_6314_MOESM2_ESM.docx]

**Supplementary Material Legend:**

**Supplementary Material Page 1: Competency Assessment Tool (CAT) for Microsurgery**

Microsurgical CAT including the parameters of surgical performance: Vascular dissection (Q1.1), Tissue preserving technique (Q1.2), Distance of suture to vessel’s margin (Q1.3), Needle holder and forceps handling (Q2.1), Needle handling (Q2.2), Thread handling/ knot tying (Q2.3), Seam quality reverse side (Q3.1), Time needed for procedure (Q4.1) and Perfusion test (Q4.2). The scores for each level are shown in brackets. Higher scores correspond to better performance.

**Supplementary Material Page 2: Assessor rating: Graphic rating scale**

The following graphical representation depicts the previously described assessors' evaluation from the microsurgical CAT, as detailed in **Table 1**. In Q5.1 to 5.3, the rater summarized thematic groups of the previous questions using graphical rating scales. Higher scores correspond to better performance.

**Supplementary Material Page 3: Self-assessment on the conventional chicken model**

The self-assessment questionnaire was administered to pre- and postgraduate students following each course day, with the objective of gauging their perceptions of the conventional chicken model. Higher scores on the questionnaire indicate a more favorable response.

**Supplementary Material Page 4: Self-assessment on the head and neck RACE model**

The self-assessment questionnaire was administered to pre- and postgraduate students following each course day (after introducing the head and neck RACE model), in order to measure their perception of the head and neck RACE model. Higher scores on the questionnaire indicate a more favorable response.

**Supplementary Material Page 5: Author information**

Author information including article title, journal name, author names; affiliation and e-mail address of the corresponding author.
